# Supplementary material for: Identification of photoperiod-induced specific miRNAs in the adrenal glands of Sunite sheep (Ovis aries)
Source: Front Vet Sci. 2022 Jul 22;9:888207. doi: 10.3389/fvets.2022.888207 (PMC9354845; doi:10.3389/fvets.2022.888207)
Supplement: Supplementary file 1 [file Data_Sheet_1.ZIP › Supplementary file/miRDeep2 file.zip!/miRDeep2 file/Know_Structure/SPLP42A1/oar-mir-3959.pdf]

miRBase precursor : oar-mir-3959
Total read count : 21079
oar-miR-3959-5p read count : 18770
oar-miR-3959-3p read count : 2308
remaining reads : 1

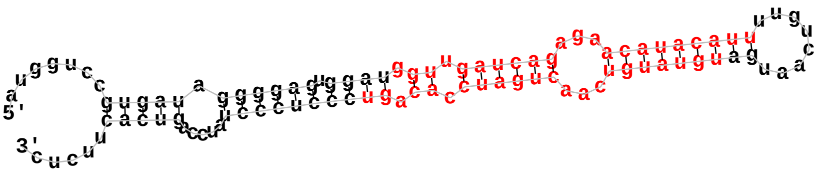

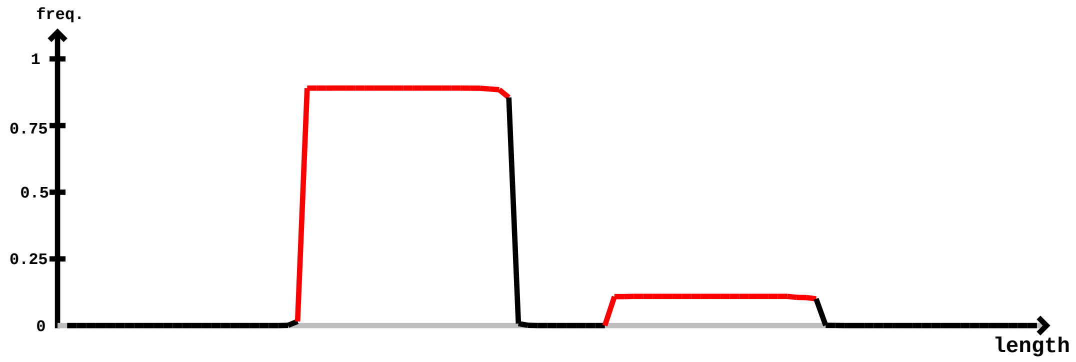

oar-miR-3959-5p

oar-miR-3959-3p

```
5'- augguccgugauaggggaguggaugguugaucagagaacauacauuuugucaaugauguaugucaacugauccacagucccucccuauccagucacuucuc   -3'    exp
    .......(((((.((((((.((((.((.((((((...(((((((((........)))))))))...)))))).)).))))))))))).....)))))..... reads     mm      sample
    ......................augguugaucagagaacau............................................................ 1         0       seq
    ......................augguugaucagagaacaua........................................................... 1         0       seq
    ......................augguugaucagagaacauac.......................................................... 7         0       seq
    ......................augguugaucagagaacauaca......................................................... 7         0       seq
    ......................augguugaucagagaacauacauu....................................................... 1         0       seq
    .......................ugguugaucagagaacaua........................................................... 1         0       seq
    .......................ugguugaucagagaacauac.......................................................... 4         0       seq
    .......................ugguugaucagagaacauaca......................................................... 4         0       seq
    .......................ugguugaucagagaacauacau........................................................ 17        0       seq
    .......................ugguugaucagagaacauacauu....................................................... 282       0       seq
    .......................ugguugaucagagaacauacauuu...................................................... 1         0       seq
    ........................gguugaucagagaacau............................................................ 4         0       seq
    ........................gguugaucagagaacaua........................................................... 5         0       seq
    ........................gguugaucagagaacauac.......................................................... 47        0       seq
    ........................gguugaucagagaacauaca......................................................... 46        0       seq
    ........................gguugaucagagaacauacau........................................................ 597       0       seq
    ........................gguugaucagagaacauacauu....................................................... 17591     0       seq
    ........................gguugaucagagaacauacauuu...................................................... 133       0       seq
    ........................gguugaucagagaacauacauuuu..................................................... 17        0       seq
    ........................gguugaucagagaacauacauuuugu................................................... 1         0       seq
    .........................guugaucagagaacauacauu....................................................... 1         0       seq
    .........................guugaucagagaacauacauuuu..................................................... 1         0       seq
    ...........................ugaucagagaacauacauuuugu................................................... 1         0       seq
    ..............................................uugucaaugauguaugucaacugauccacagu....................... 1         0       seq
    ........................................................uguaugucaacugauccac.......................... 83        0       seq
    ........................................................uguaugucaacugauccaca......................... 12        0       seq
    ........................................................uguaugucaacugauccacag........................ 100       0       seq
    ........................................................uguaugucaacugauccacagu....................... 2093      0       seq
    ........................................................uguaugucaacugauccacaguc...................... 3         0       seq
    ..........................................................uaugucaacugauccacagu....................... 4         0       seq
    ..........................................................uaugucaacugauccacaguc...................... 2         0       seq
    ..........................................................uaugucaacugauccacagucc..................... 11        0       seq
```
